# Expect the unexpected: cervical gastric-type adenocarcinoma *in situ* in a patient with long-standing Peutz-Jeghers syndrome — a case report and literature review

**DOI:** 10.3389/fmed.2026.1944455

**Published:** 2026-10-09

**Authors:** Shuo Xu, Xinjun Li, Xiangnan Zhang, Shengpu Wang

**Affiliations:** 1 Department of Gynecology, Hebei General Hospital, Shijiazhuang, China; 2 Department of Pathology, Hebei General Hospital, Shijiazhuang, China; 3 Department of Obstetrics, The Second Hospital of Hebei Medical University, Shijiazhuang, China

**Keywords:** cervical cancer screening, Gastric-type adenocarcinoma, Peutz-Jeghers Syndrome, STK11, Vaginal Discharge

## Abstract

**Background:**

Peutz-Jeghers syndrome (PJS) is an autosomal dominant disorder caused by germline mutations in the STK11 tumor suppressor gene, associated with a significantly elevated lifetime risk of various malignancies. Gastric-type endocervical adenocarcinoma (G-EAC) and its precursor lesions represent one of the most challenging gynecologic manifestations due to their “deceptively bland” appearance, HPV-independent nature, and propensity to evade routine screening, often leading to delayed diagnosis and poor prognosis.

**Case presentation:**

We report a 37-year-old woman with a 30-year history of PJS, presenting with persistent watery vaginal discharge for one year. Despite negative cervical cytology, negative HPV testing, normal colposcopic findings, and inconclusive imaging, her request for hysterectomy was initially declined due to absence of definitive malignant features. The patient subsequently sought care at a tertiary referral center in Shanghai, where post-hysterectomy pathological examination unexpectedly confirmed cervical gastric-type adenocarcinoma *in situ* (GAS-AIS).

**Conclusion:**

This case highlights a critical message: a PJS patient with persistent watery vaginal discharge may harbor gastric-type cervical glandular neoplasia despite negative cytology, negative HPV testing, apparently normal cervical examination, and inconclusive imaging. Persistent watery discharge in this specific population should be considered a “red flag” for gastric-type lesions, which may arise from lobular endocervical glandular hyperplasia (LEGH). For PJS patients with persistent unexplained vaginal discharge, we suggest that clinical suspicion — rather than cytology or colposcopy findings — should be the primary trigger for diagnostic excisional procedures. We present a comprehensive literature review of PJS-associated G-EAC and advocate for updated surveillance protocols, including random cervical biopsies or excisional procedures in symptomatic PJS patients, regardless of colposcopic findings.

## 1 Background

Peutz-Jeghers syndrome (PJS) is a rare autosomal dominant disorder first described by Peutz in 1921 and later characterized by Jeghers et al. in 1949, with an estimated prevalence of approximately 1 in 50,000 to 1 in 200,000 live births (1–3). The syndrome is defined by three core features: mucocutaneous pigmentation (primarily involving the lips, oral mucosa, fingers, and toes), gastrointestinal hamartomatous polyposis, and a markedly elevated lifetime risk of both gastrointestinal and extraintestinal malignancies (4, 5). The underlying genetic basis is germline mutation in the STK11 gene (also known as LKB1), located on chromosome 19p13.3, which encodes a serine/threonine kinase that functions as a key regulator of cell polarity, energy metabolism, and mTOR signaling (6–8).

The cancer risk in PJS is substantial. A landmark study by Giardiello et al. demonstrated that by age 65 years, affected individuals have a cumulative cancer risk exceeding 90%, with a hazard ratio of 15.2 compared with the general population (9). A comprehensive systematic review by van Lier et al. confirmed these findings and established the foundation for current surveillance recommendations (5). While gastrointestinal malignancies (colorectal, gastric, pancreatic, and small bowel cancers) have received considerable attention, recent evidence indicates that the risk of gynecologic cancers, particularly those of the cervix and ovary, is significantly elevated and often underestimated (10, 11). Female PJS patients have an estimated lifetime risk of 10% for cervical cancer and 21% for ovarian cancer (12, 13).

Cervical gastric-type adenocarcinoma (G-EAC) is a rare, HPV-independent subtype of endocervical adenocarcinoma that accounting for approximately 10% of all cervical adenocarcinomas in some series (14–16), though the proportion varies across populations, but is disproportionately overrepresented in PJS patients (11). Unlike the more common usual-type endocervical adenocarcinoma (UEA) — which is strongly associated with high-risk HPV and typically presents with visible exophytic lesions — G-EAC is notorious for its “deceptively bland” histology, lack of cytologic abnormalities on routine Pap smears, and propensity for superficial spread beneath an intact cervical epithelium (14).

The precursor -associated lesion of G-EAC is believed to be lobular endocervical glandular hyperplasia (LEGH), a gastric-type mucinous glandular proliferation that represents the putative precursor-associated lesion in the LEGH–GAS-AIS–G-EAC sequence; however, malignant transformation is uncommon, with reported rates of approximately 1–2% (17, 18). In a cohort of 135 patients with cervical lesions, 63.0% of those with suspected LEGH presented with watery discharge, underscoring the symptom's diagnostic significance (19). Importantly, LEGH and early G-EAC may produce profuse watery or mucoid vaginal discharge — often the sole presenting symptom — while the cervix appears normal or only mildly hypertrophic on clinical examination (20).

We present a case of cervical GAS-AIS in a 37-year-old woman with long-standing PJS, whose initial request for hysterectomy was declined due to the absence of definitive malignant findings. This case serves as a cautionary tale and provides an opportunity to review the current literature on PJS-associated cervical gastric-type neoplasia, with emphasis on clinical recognition, molecular pathogenesis, and implications for surveillance.

## 2 Case description

### 2.1 Patient history

A 37-year-old woman with a 30-year history of Peutz-Jeghers syndrome presented to our gynecology department with a chief complaint of profuse, clear, watery vaginal discharge for one year. She also reported irregular menstrual cycles (30-60 days) and dysmenorrhea requiring analgesic medication. Her last menstrual period was March 4, 2026. She is gravida 1, para 1.

Her past medical history was notable for extensive surgical interventions related to PJS. She had undergone six laparotomies for intestinal obstruction (at ages 19, 25, 27, and 29 years; two additional procedures in early childhood, exact ages unknown), with segmental small bowel resections. She had also undergone three colonoscopic polypectomies at ages 29, 32, and 35 years. At age 30 years, she was diagnosed with stage IA lung papillary adenocarcinoma and underwent curative lung segmentectomy. At age 35 years, she underwent right adnexectomy for an ovarian mass, with postoperative pathology revealing benign Brenner tumor with mucinous cystadenoma. Family history was notable for her father having died of liver cancer; however, formal genetic testing of family members had not been performed.

### 2.2 Initial presentation and diagnostic workup

On admission, physical examination was unremarkable except for characteristic mucocutaneous pigmentation on the lips and digits. Vital signs were stable. Abdominal examination revealed multiple well-healed surgical scars with a soft, non-tender abdomen. Pelvic examination revealed a normal-sized uterus and adnexa. Speculum examination showed a smooth but mildly hypertrophic cervix with copious clear fluid emanating from the external os. No exophytic lesions, ulcerations, or visible masses were appreciated. Bimanual examination revealed no cervical motion tenderness or adnexal masses.

Laboratory studies revealed mildly elevated CA-125 (41.8 U/mL) and normal HE4 (53.8 pmol/L). Cervical cytology (Pap smear) was negative for intraepithelial lesions or malignancy (NILM). HPV E6/E7 mRNA testing was negative, confirming the HPV-independent nature of the lesion. Pelvic MRI demonstrated a mildly thickened endometrium and a lesion in the right anterior fundal myometrium suggestive of adenomyosis. The cervix appeared unremarkable except for nabothian cysts. A left adnexal cystic lesion (O-RADS 3) was noted. Notably, no distinct cervical mass was identified. On retrospective re-review, subtle multicystic changes in the cervix were noted, which were consistent with the characteristic appearance of LEGH (Figure 1).

**Figure 1:**
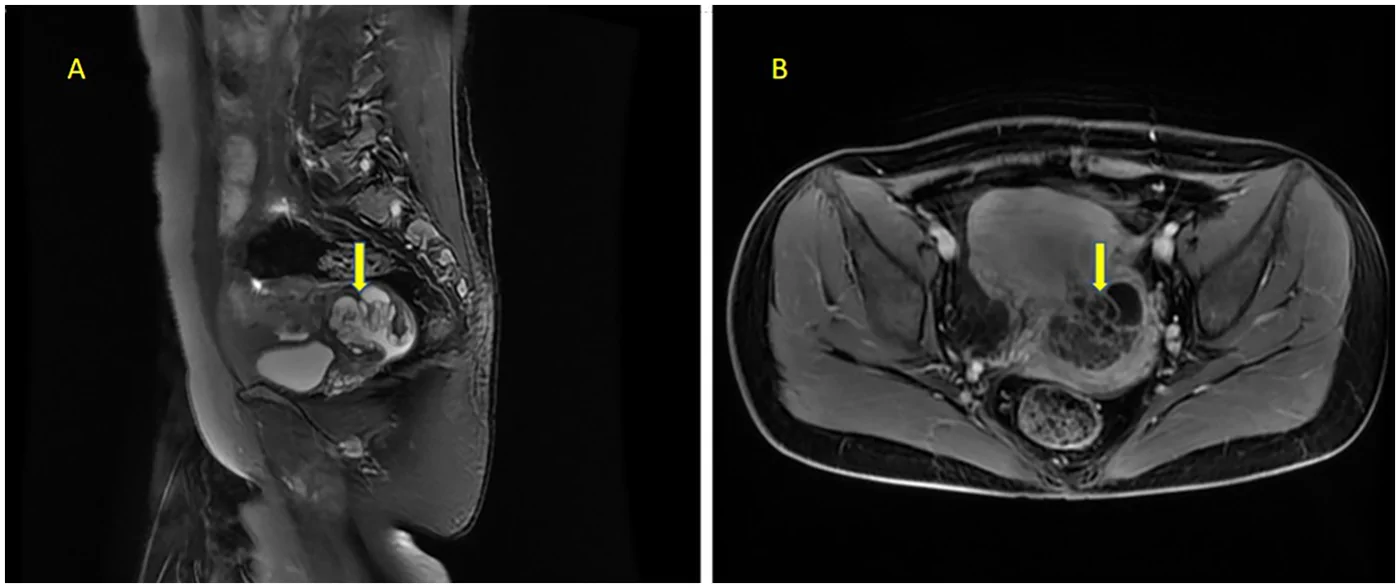
Pelvic MRI findings. **(A)** T2-weighted sagittal image showing multicystic lesions in the cervix (arrow). **(B)** Contrast-enhanced axial image demonstrating the multicystic appearance of the cervix (arrow), which is characteristic of lobular endocervical glandular hyperplasia (LEGH).

### 2.3 Clinical decision-making and initial decline of hysterectomy

A multidisciplinary discussion was held to determine the appropriateness of hysterectomy. The diagnostic workup was challenging due to the normal Pap smear, negative HPV testing, unremarkable imaging, and absence of visible cervical lesions despite persistent symptoms. Given the absence of a visible cervical lesion, negative cytology, and lack of definitive imaging evidence for malignancy, the gynecologic oncology team concluded that the indication for hysterectomy was insufficient. The patient's request for definitive surgical management was therefore declined, and she was counseled regarding expectant management with close surveillance. In retrospect, this decision reflected a discrepancy between current screening guidelines and the unique biology of PJS-associated cervical neoplasia, rather than a clinical error per se.

### 2.4 Subsequent management and definitive diagnosis

Dissatisfied with this approach, the patient sought a second opinion at a tertiary referral center in Shanghai. Based on the combination of PJS diagnosis, persistent watery discharge, and mild cervical hypertrophy—despite negative Pap and imaging—the consulting team recommended diagnostic excisional procedure. The patient was counseled regarding the risks and benefits and agreed to proceed. The patient underwent total hysterectomy with left salpingo-oophorectomy (the right ovary had been previously removed at age 35).

Postoperative pathological examination revealed:

- Cervix: Gastric-type adenocarcinoma *in situ* (GAS-AIS) arising in association with lobular endocervical glandular hyperplasia (LEGH); negative surgical margin
- Uterus: Diffuse adenomyosis; endometrial polyp with early secretory phase endometrium
- Left ovary: Mucinous cystadenoma with gastric-type differentiation; associated with multifocal sex cord tumor with annular tubules (SCTAT)
- Left fallopian tube: No pathological changes
- No evidence of invasive carcinoma or extrauterine spread

The H&E slides were obtained from the Department of Pathology of a tertiary hospital in Shanghai and reviewed by our senior pathologist. Representative photomicrographs are shown in Figure 2. The entire cervix was serially sectioned at 3-mm intervals; AIS without invasion was confirmed by absence of stromal invasion, intact basement membranes, and negative margins. Immunohistochemical studies demonstrated gastric-type mucin phenotype (CK7+, MUC6+, Claudin18.2+, ER−, PR−, p16 patchy+, p53 wild-type, Ki-67 30% in the *in situ* component). IHC slides were not available due to the outside hospital's policy; the electronic report is included as Supplementary Material.

**Figure 2:**
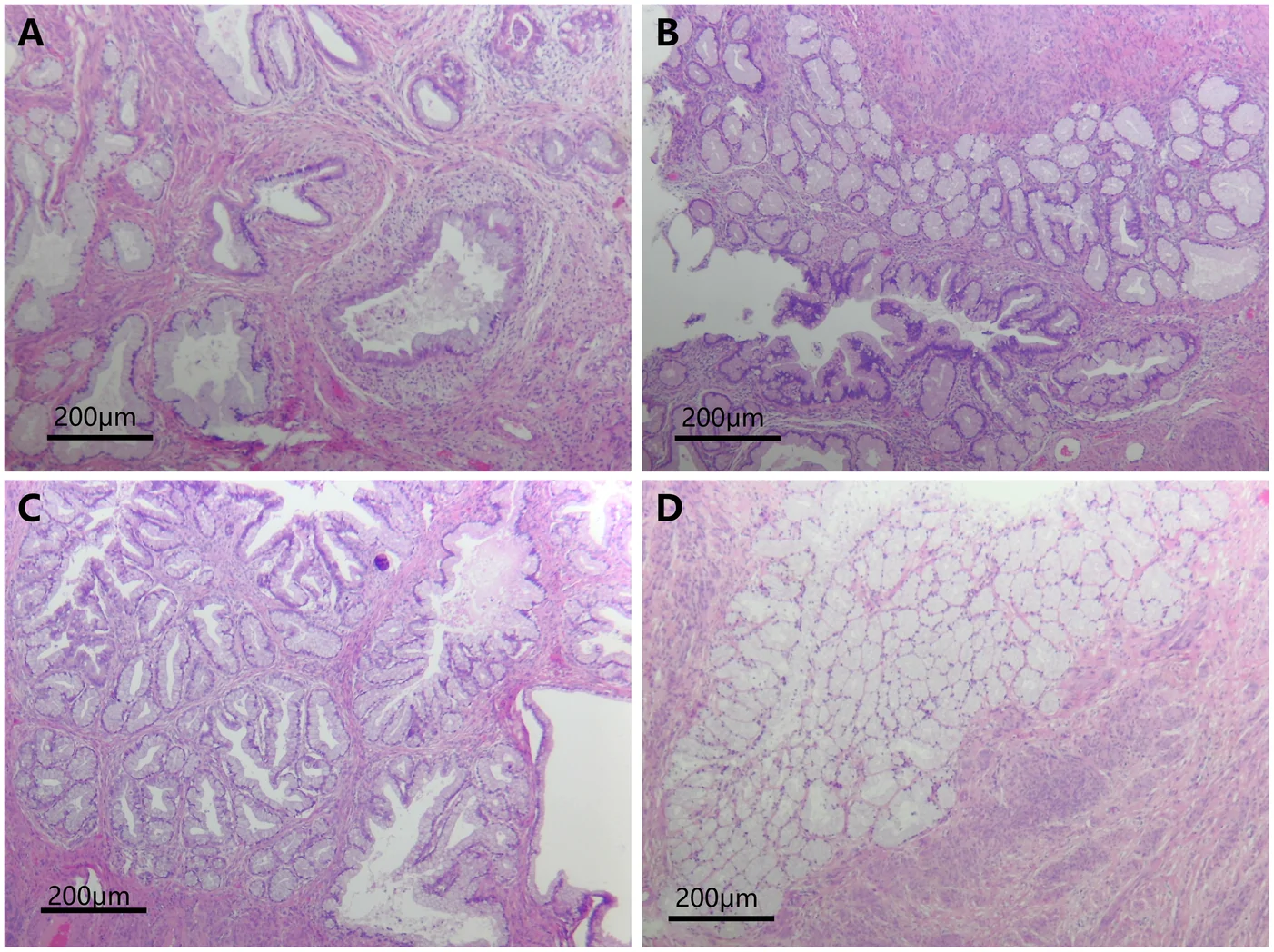
Histopathological findings. **(A)** Low-power view showing lobular endocervical glandular hyperplasia (LEGH) with architectural complexity (H&E, ×40). **(B)** High-power view of LEGH with gastric-type mucinous epithelium (H&E, ×200). **(C)** Gastric-type adenocarcinoma *in situ* (GAS-AIS) showing nuclear stratification and hyperchromasia (H&E, ×200). **(D)** High-power view of the *in situ* component with cytological atypia and intact basement membrane (H&E, ×400).

### 2.5 Follow-up

The patient recovered uneventfully from surgery. At six-month follow-up, she remained asymptomatic with no evidence of residual or recurrent disease on pelvic examination and imaging. She reported complete resolution of the preoperative vaginal discharge and expressed satisfaction with the surgical outcome. The patient was informed of the potential benefits of germline STK11 sequencing. After thorough counseling, she declined genetic testing due to financial constraints, as the test was not covered by her insurance. The clinical diagnosis of PJS, established over 30 years ago based on classic features, remains robust given her consistent long-term clinical course. She has been referred for ongoing surveillance for other PJS-associated malignancies, including breast, pancreatic, and colorectal cancers. The patient expressed relief at having a definitive diagnosis and resolution of her symptoms, and hoped that her experience would encourage clinicians to take persistent symptoms seriously in PJS patients (Figure 3).

**Figure 3:**
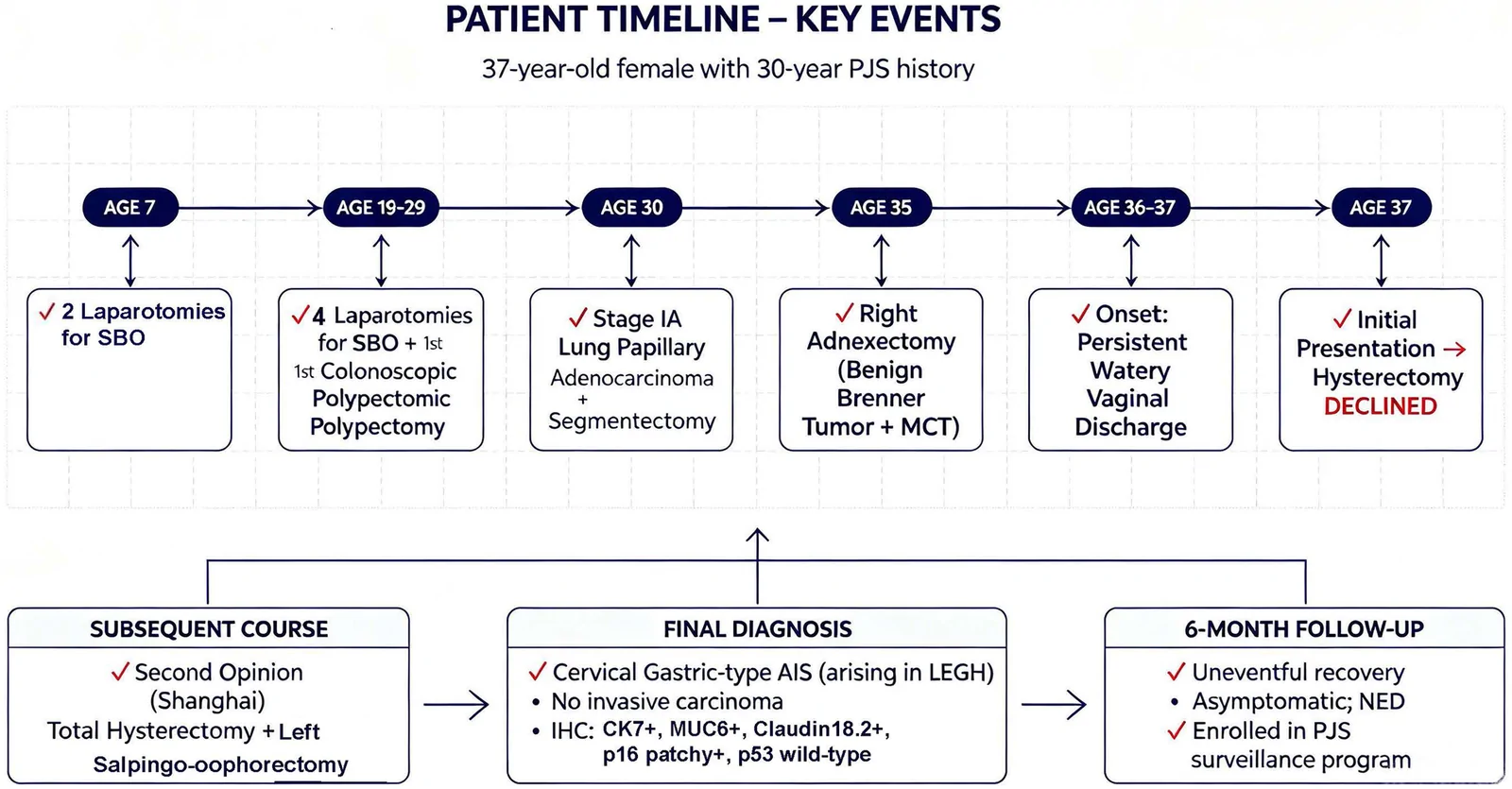
Patient timeline. This 37-year-old female with a 30-year history of Peutz-Jeghers syndrome (PJS) experienced multiple PJS-related surgical interventions beginning in childhood, including six laparotomies for small bowel obstruction, three colonoscopic polypectomies, lung segmentectomy for stage IA papillary adenocarcinoma at age 30, and right adnexectomy (benign Brenner tumor with mucinous cystadenoma) at age 35. At age 36, she developed persistent watery vaginal discharge, which prompted her initial presentation. Despite negative cytology and imaging, her request for hysterectomy was initially declined. She subsequently underwent total hysterectomy with left salpingo-oophorectomy in Shanghai. Final pathology revealed GAS-AIS arising in LEGH. At six-month follow-up, she remained disease-free.

## 3 Discussion

For the literature review component of this report, we searched PubMed, Web of Science, and Embase for English-language publications up to 2026 using the following search terms: ‘Peutz-Jeghers syndrome,’ ’STK11,’ ‘gastric-type adenocarcinoma,’ ‘cervical,’ and ‘lobular endocervical glandular hyperplasia.’ Reference lists of relevant articles were also screened for additional publications.

### 3.1 Stk11 mutation and molecular pathogenesis of PJS-associated neoplasia

Peutz-Jeghers syndrome is caused by heterozygous germline mutations in the STK11 gene (serine/threonine kinase 11, also known as LKB1), located on chromosome 19p13.3 (6, 7). STK11 encodes a 50-kDa serine/threonine kinase that functions as a tumor suppressor through multiple interconnected signaling pathways (21). The protein is a master regulator of cellular energy homeostasis, acting upstream of AMPK (AMP-activated protein kinase) and the 12 other AMPK-related kinases (22, 23). Through these effectors, STK11 controls cell polarity, growth arrest, apoptosis, and metabolism (24).

Two-hit inactivation of STK11 — germline mutation followed by somatic loss of heterozygosity — is the classical mechanism of tumorigenesis in PJS (25). Somatic STK11 mutations have also been identified in sporadic cancers, including lung, pancreatic, and cervical carcinomas (26–28). Recent studies have demonstrated that STK11 loss leads to mTOR pathway activation via TSC2 inactivation, promoting cell growth and proliferation (29, 30).

Importantly, STK11 plays a critical role in the response to immune checkpoint blockade. Pons-Tostivint et al. comprehensively reviewed that STK11-mutant tumors, particularly in lung cancer, exhibit an immunosuppressive tumor microenvironment characterized by reduced T-cell infiltration and resistance to anti-PD-1 therapy (31). Whether this observation extends to PJS-associated cervical cancers remains an area of active investigation.

A recent bibliometric analysis by Niu et al. mapped global research trends in PJS-associated gynecologic cancers, identifying a growing focus on STK11 molecular pathogenesis and the need for improved surveillance strategies (11). This analysis revealed only 96 English-language publications on the topic from 2010 to 2024, underscoring the rarity of the condition and the associated challenges in developing evidence-based guidelines.

While formal germline STK11 testing was not performed in this patient due to financial constraints, her clinical presentation—including characteristic mucocutaneous pigmentation, recurrent gastrointestinal polyposis requiring multiple surgical interventions, and family history of malignancy—is fully consistent with the diagnosis of PJS. The link between STK11 mutation and gastric-type cervical neoplasia is well-established in the literature, and our case adds important clinical weight to this association by illustrating the diagnostic challenges posed by these lesions. We acknowledge the absence of molecular confirmation as a limitation of this report, and encourage clinicians to pursue genetic testing when feasible in similar cases.

Beyond the classical two-hit inactivation, oncogenic alterations such as KRAS mutation and aberrant p16 expression have also been implicated in the pathogenesis of PJS-associated G-EAC, suggesting a multistep process of carcinogenesis beyond STK11 loss alone (32). Furthermore, STK11 mutations in G-EAC have been linked to poor prognosis, with one study demonstrating that STK11 mutations were significantly associated with worse survival outcomes (*p* = 0.01) (33).

### 3.2 The LEGH-AIS-G-EAC continuum: a distinct pathway of cervical carcinogenesis

Gastric-type endocervical adenocarcinoma (G-EAC) is recognized as a distinct entity in the 2020 World Health Organization classification of female genital tumors (34). Unlike usual-type endocervical adenocarcinoma (UEA), which is driven by high-risk HPV and characterized by p16 block positivity, G-EAC is HPV-independent and exhibits a gastric immunophenotype (MUC6+, MUC5AC+, HIK1083+) (15).

The precursor of G-EAC is believed to be lobular endocervical glandular hyperplasia (LEGH), a lesion composed of pyloric-type gastric glands (17, 35). LEGH can progress through gastric-type adenocarcinoma *in situ* (GAS-AIS) to frankly invasive G-EAC. This continuum has been termed the “LEGH-AIS-G-EAC sequence” and shares molecular similarities with gastric carcinogenesis (36, 37). However, the frequency of malignant transformation of LEGH during surveillance appears limited, with one longitudinal study reporting a rate of 1.4% (1/69 patients) over a mean follow-up of 57.1 months (18). Notably, worsening cytology and increasing lesion size were identified as important parameters for detecting malignant transformation (18), highlighting the importance of active surveillance in symptomatic patients.

A critical clinical implication of this pathway is that both LEGH and early G-EAC may produce abundant watery or mucoid vaginal discharge—often the sole presenting symptom—while the cervix appears normal on speculum examination (32). In a comprehensive review by Miyamoto et al., the authors emphasized that LEGH is not merely a benign metaplasia but a precursor-associated lesion a with a reported malignant transformation rate of 1%–2% (17).

Our case exemplifies this diagnostic challenge. The patient's cervix was described as “smooth” and “hypertrophic” but without visible lesions. Yet the copious discharge—a feature she had experienced for one year—was a critical clue that was initially underappreciated. The presence of LEGH adjacent to AIS in the final pathology specimen supports the precursor-product relationship.

### 3.3 Why routine screening fails: limitations of Pap smear and HPV testing

Conventional cervical cancer screening paradigms are ineffective for detecting gastric-type lesions. This failure stems from three interrelated factors.

First, G-EAC is not associated with HPV infection. Multiple studies have confirmed that HPV DNA is absent or detected at very low rates (<10%) in G-EAC, compared with >90% in UEA (16, 38). Consequently, HPV co-testing—which has dramatically improved the sensitivity of screening for HPV-associated disease—offers no benefit for detecting gastric-type neoplasia.

Second, cytologic sampling frequently misses gastric-type lesions. The neoplastic glands are located deep within the cervical stroma, often beneath an intact squamous epithelium, and the cells shed poorly into the endocervical canal (39, 40). A systematic review by Gordhandas et al. found that among PJS patients with confirmed G-EAC, 50% had normal Pap smears prior to diagnosis (41). This alarming statistic underscores the inadequacy of cytology-based screening in this population.

Third, colposcopic appearance is deceptive. G-EAC lacks the acetowhite change and atypical vessels characteristic of HPV-associated lesions. Instead, the cervix may appear normal, mildly hypertrophic, or exhibit a “cobblestone” pattern (42, 43).

These limitations have important implications for PJS surveillance. Current guidelines from the National Comprehensive Cancer Network (NCCN) recommend annual pelvic examination and Pap smear beginning at age 18-20 for PJS patients (44). However, accumulating evidence suggests this approach is inadequate for detecting gastric-type lesions (11, 41). The systematic review by Gordhandas et al. concluded that improved screening strategies, including a low threshold for imaging and biopsy, are urgently needed for this high-risk population (41).

### 3.4 Clinicopathological features and prognosis of G-EAC

Gastric-type endocervical adenocarcinoma carries a significantly worse prognosis than usual-type adenocarcinoma, independent of stage (45, 46). Several clinicopathological differences account for this aggressive behavior.

G-EAC exhibits deeper invasion, larger horizontal spread, more frequent parametrial and vaginal extension, and higher rates of lymph node metastasis at diagnosis compared to stage-matched UEA (47, 48). The aggressive nature of G-EAC is further underscored by clinical data: in a cohort of 15 G-EAC patients, 60% presented at advanced stage (FIGO III/IV), two patients died within 10 months postoperatively, and five developed distant metastasis (49). The poor prognosis is particularly pronounced in PJS patients, who are predisposed to early-onset and chemoresistant disease (11, 41).

The poor prognosis is partly explained by late diagnosis—a direct consequence of the diagnostic challenges discussed above. However, intrinsic biological aggressiveness also plays a role. A case report by Wu et al. described a 32-year-old PJS patient with cervical G-EAC that extensively metastasized to the bilateral ovaries, endometrium, fallopian tubes, and pelvic cavity; next-generation sequencing revealed a CDKN2A frameshift mutation in addition to STK11 alteration (50). The patient died just 4 months after completing chemotherapy, underscoring the aggressive potential of these tumors.

Recent genomic analyses have shed light on the molecular landscape of G-EAC. A large-cohort study of 100 patients with gastric-type adenocarcinoma (GAS)(median age 50 years) identified a distinct somatic mutation profile, with TP53 (72.7%), KRAS (45.5%), SMAD4 (45.5%), CDKN2A (36.4%), PIK3CA (27.3%), and STK11 (18.2%) being the most frequently altered genes, showing molecular homology with pancreaticobiliary adenocarcinoma (51). This profile aligns with findings from other comprehensive studies of HPV-independent cervical adenocarcinomas, which have similarly identified TP53 as the most frequently mutated gene, followed by MSH6, CDKN2A/B, POLE, SLX4, ARID1A, STK11, and BRCA2 (52). Notably, HER2 amplification has been detected in a subset of G-EACs, suggesting potential therapeutic opportunities for anti-HER2 therapy (53, 54). Furthermore, PD-L1 expression (CPS ≥ 1) was significantly associated with shorter progression-free survival (*p* = 0.046) in the same cohort, suggesting potential immunotherapy opportunities for a subset of patients (51).

### 3.5 Synchronous mucinous lesions and the field effect

A particularly concerning phenomenon in PJS patients is the development of synchronous mucinous metaplasia and neoplasia of the female genital tract (SMMN-FGT), a rare condition characterized by multifocal mucinous lesions involving at least two sites among the cervix, endometrium, fallopian tubes, and ovaries (55, 56). SMMN-FGT appears to be overrepresented in PJS patients due to the underlying STK11 mutation affecting Müllerian-derived epithelia.

Our patient did not meet criteria for SMMN-FGT as mucinous lesions were confined to the cervix. However, the presence of endometrial polyps with mucinous metaplasia raises the possibility of a field effect, and long-term surveillance remains essential.

### 3.6 Proposed revision of surveillance protocols

Based on the accumulating evidence, several authors have called for revised surveillance protocols for PJS patients, particularly for cervical cancer screening (41). However, we acknowledge the low malignant transformation rate of LEGH (1.4% reported by Kobara et al. 18), and we emphasize that our recommendations are intended to inform shared decision-making rather than mandate universal intervention. Therefore, we present the following as proposed considerations for multidisciplinary discussion, particularly for symptomatic PJS patients. Proposed modifications based on the literature include:

1. Lowered threshold for diagnostic excisional procedures: Any PJS patient with persistent unexplained vaginal discharge or gross cervical hypertrophy should undergo diagnostic excisional procedure [loop electrosurgical excision procedure (LEEP) or cold knife conization (CKC)], regardless of cytology and colposcopy findings (41, 50). This principle is further supported by recent reports demonstrating that even patients lacking classic PJS features but carrying mosaic STK11 mutations may present with identical symptoms and be diagnosed with GAS-AIS only after LEEP prompted by clinical suspicion (57).
2. Random cervical biopsies: For symptomatic patients without visible lesions, random biopsies from the transformation zone and endocervical curettage may detect early gastric-type lesions (11, 41).
3. Annual pelvic MRI consideration: Given that G-EAC often presents with a “cystic” appearance on imaging, adding annual pelvic MRI to the surveillance protocol may improve early detection of cervical and ovarian pathology (17, 42, 58). The MRI findings in our case, initially interpreted as unremarkable, demonstrated on retrospective review a multicystic appearance characteristic of LEGH, underscoring the importance of careful imaging evaluation in symptomatic PJS patients.
4. Low threshold for hysterectomy: While prophylactic hysterectomy remains controversial, symptomatic PJS patients with completed childbearing should be counseled regarding the potential benefits of definitive surgery (12, 47, 51).
5. Multidisciplinary surveillance: Given the spectrum of PJS-associated malignancies (breast, pancreatic, colorectal, and gynecologic), coordinated multidisciplinary surveillance is essential (3, 59, 60).

### 3.7 Strengths and limitations

This case has several strengths. First, it provides a detailed clinical trajectory from initial presentation to definitive diagnosis, highlighting a common but underappreciated clinical pathway. Second, it offers a comprehensive literature review synthesizing recent advances in the field. Third, it emphasizes the critical importance of clinical symptoms over normal screening tests in PJS patients. Fourth, the diagnosis was independently confirmed by slide review by our senior pathologist.

Limitations include the inherent constraints of a single case report, which precludes generalizability. Additionally, long-term follow-up data are not yet available to assess oncologic outcomes. Immunohistochemical images are not available due to the institutional policy of the outside hospital; the complete IHC results are documented in the electronic report (Supplementary Material).

## 4 Conclusions

This case report and comprehensive literature review underscore a critical message: a PJS patient with persistent watery vaginal discharge may harbor gastric-type cervical glandular neoplasia despite negative cytology, negative HPV testing, apparently normal cervical examination, and inconclusive imaging.

First, persistent watery vaginal discharge in a PJS patient should be considered a “red flag” for gastric-type endocervical neoplasia, even when the cervix appears normal and Pap smears are negative. Second, routine cervical cancer screening—including HPV co-testing—is inadequate for detecting gastric-type lesions, which are HPV-independent and cytologically elusive. Third, the threshold for diagnostic excisional procedures in symptomatic PJS patients must be lowered; a “smooth cervix” does not exclude high-grade glandular neoplasia. Fourth, pathologists should maintain a high index of suspicion for gastric-type lesions in any cervical specimen from a PJS patient, employing appropriate mucin immunohistochemistry (MUC6, MUC5AC, HIK1083) to confirm the diagnosis.

Our patient was initially denied surgery due to insufficient evidence—a decision that, in retrospect, highlighted a critical gap between current screening guidelines and the unique biology of PJS-associated cervical neoplasia. We present this case to prevent similar missed or delayed diagnoses in the future.

As our understanding of STK11 biology and gastric-type carcinogenesis continues to evolve, we anticipate the development of improved screening strategies and, potentially, targeted therapies for this challenging group of patients.

## Data Availability

The original contributions presented in the study are included in the article/Supplementary Material, further inquiries can be directed to the corresponding author.
