# Supplementary material for: Expect the unexpected: cervical gastric-type adenocarcinoma in situ in a patient with long-standing Peutz-Jeghers syndrome — a case report and literature review
Source: Front Med (Lausanne). 2026 Oct 9;13:1944455. doi: 10.3389/fmed.2026.1944455 (PMC13692713; doi:10.3389/fmed.2026.1944455)
Supplement: Supplementary file 1 [file Datasheet1.pdf]

CARE Checklist of information to include when writing a case report

(cc) BY-NC-ND

Topic	Item	Checklist item description	Reported on Line
Title	1	The diagnosis or intervention of primary focus followed by the words "case report"	1-3
Key Words	2	2 to 5 key words that identify diagnoses or interventions in this case report, including "case report"	13-14
Abstract (no references)	3a	Introduction: What is unique about this case and what does it add to the scientific literature?	16-21
	3b	Main symptoms and/or important clinical findings	22-23
	3c	The main diagnoses, therapeutic interventions, and outcomes	25-27
	3d	Conclusion—What is the main "take-away" lesson(s) from this case?	28-37
Introduction	4	One or two paragraphs summarizing why this case is unique (**may include references**)	74-78
Patient Information	5a	De-identified patient specific information	81
	5b	Primary concerns and symptoms of the patient	82
	5c	Medical, family, and psycho-social history including relevant genetic information	85-119
	5d	Relevant past interventions with outcomes	121-126
Clinical Findings	6	Describe significant physical examination (PE) and important clinical findings	95-109
Timeline	7	Historical and current information from this episode of care organized as a timeline	Figure 1
Diagnostic Assessment	8a	Diagnostic testing (such as PE, laboratory testing, imaging, surveys)	102-109
	8b	Diagnostic challenges (such as access to testing, financial, or cultural)	111-119
	8c	Diagnosis (including other diagnoses considered)	121-126
	8d	Prognosis (such as staging in oncology) where applicable	145-155
Therapeutic Intervention	9a	Types of therapeutic intervention (such as pharmacologic, surgical, preventive, self-care)	121-126
	9b	Administration of therapeutic intervention (such as dosage, strength, duration)	121-126
	9c	Changes in therapeutic intervention (with rationale)	121-126
Follow-up and Outcomes	10a	Clinician and patient-assessed outcomes (if available)	145-155
	10b	Important follow-up diagnostic and other test results	145-155
	10c	Intervention adherence and tolerability (How was this assessed?)	153-155
	10d	Adverse and unanticipated events	129-130
Discussion	11a	A scientific discussion of the strengths AND limitations associated with this case report	310-318
	11b	Discussion of the relevant medical literature **with references**	157-308
	11c	The scientific rationale for any conclusions (including assessment of possible causes)	157-308
	11d	The primary "take-away" lessons of this case report (without references) in a one paragraph conclusion	302-339
Patient Perspective	12	The patient should share their perspective in one to two paragraphs on the treatment(s) they received	153-155
Informed Consent	13	Did the patient give informed consent? Please provide if requested	**Yes**
